# Integrating student feedback during “Dental Curriculum Hack-A-thon”

**DOI:** 10.1186/s12909-018-1189-z

**Published:** 2018-05-02

**Authors:** Shawheen S. Saffari, R. Frederick Lambert, Lucy Dang, Sarah Pagni, Irina F. Dragan

**Affiliations:** 1 Department of Dentistry & Oral Surgery, St. Barnabas Hospital, Bronx, USA; 2 Harvard School of Dental Medicine, Boston, USA; 3 Boston University Henry M. Goldman School of Dental Medicine, Boston, USA; 4 Department of Public Health and Community Service, Tufts University School of Dental Medicine, Boston, USA; 5 Department of Periodontology, Tufts University School of Dental Medicine, One Kneeland Street, Boston, MA 02111 USA

**Keywords:** Dental education, Curriculum innovation, Student preferences

## Abstract

**Background:**

The future of dental education is at crossroads. This study used the parameter of the 2016 Dental Curriculum Hack-a-Thon to assess intra- and inter-institutional agreement between student and faculty views regarding dental curriculums to determine if there is an impact in student perceptions towards dental education from before and after the event.

**Methods:**

This exploratory, cross-sectional study involved two surveys, with Survey 1 being distributed among four faculty-student pairs of the four participating dental schools answering 14 questions. Survey 2 assessed the views of 20 participating dental students through 26 questions in a pre- and post- event survey design. Descriptive statistics were used to explore differences in perceptions towards dental education across both instrument surveys.

**Results:**

The results revealed valuable student insights regarding intra- and inter-institutional agreement relevant for the change in dental curriculum that needs to occur. Survey 2 revealed that mandatory attendance in didactic courses, electronic based examination preferences, and the preference of preclinical courses being held in the first and second years of a four-year dental curriculum were of particular importance to student participants.

**Conclusions:**

The results of this study indicate that exposure and participation in subjects pertaining to dental education can be influential on student preferences and opinions on how dental education should be delivered in a four-year curriculum.

## Background

The current climate in health care is one of reform. Dentistry is not separate from this movement and moreover, dental education should remain a major focus of reform. It is with high standards of scholarship, research, and education that the dental profession can remain at the forefront of quality oral health care delivery. Ensuring the highest standards of education allows patients to receive the highest quality of care. With the changing landscapes of medical education and healthcare delivery, it is imperative that the dental community examines its field not as a separate entity but one included in holistic patient care [1, 2].

In 1926, the Gies report was the first comprehensive review of dental education and made several recommendations for reform [3, 4]. A major thrust of this report was the elevation of dental schools to the same quality and standards of medical schools and universities. Since then, a variety of publications addressed dental education reform [5, 6]. The first to include dental education consideration, was a report by the Institute of Medicine (IOM) entitled “Dental Education at the Crossroads: Challenges and Change” [7]. The Surgeon General of the United States of America Report discussed the growing access to dental care problem in the U.S. and its relationship with dental education. The report describes a critical need for dental researchers and faculty members to meet the demands of the country’s oral healthcare needs [8]. The IOM report also urged continued testing of alternative models of education, practice, and performance assessment for dentists and allied dental professionals which is necessary to prepare the dental community – educators, practitioners, regulators, and policy makers for an uncertain future.

The increasing use of technology, changing pedagogy and soaring costs of dental education have also sparked change [9–11]. Some reformation strategies include reduce the number of large group lectures and emphasize the integration of technology and different systems for delivering information, such as flipped classroom, small group discussions, and case-based learning [12–15]. Education literature supports the use of these methods in the improved delivery and retention of information [16].

While the movement toward educational reform has already begun, little is known about the effect of student involvement in curricular development. Recently, the student chapters of American Dental Education Association (ADEA) at Boston University Henry M. Goldman School of Dental Medicine (BUGSDM) Harvard School of Dental Medicine (HSDM), Tufts University School of Dental Medicine (TUSDM) and University of New England College of Dental Medicine (UNECDM) met for the “New England Dental Curriculum Hack-a-Thon” initiative. The “Hack-a-thon” terminology was inspired from computer programming (https://en.wikipedia.org/wiki/Hackathon). This can be an educational and social event when participants work collaboratively with a focus on a particular topic and develop innovative projects. At the end of the day/event, each team presents outcomes and often times experts in the field are providing feedback on the feasibility of the project. The “New England Dental Curriculum Hack-a-Thon” event brought pre-doctoral students, post-doctoral students, and faculty from four different New England dental schools together in an integrated way to discuss and debate dental curriculum development. To the best of our knowledge a comprehensive curriculum design program that incorporates student feedback directly has not yet been reported.

The aim of this exploratory study was to assess intra- and inter-institutional agreement between student and faculty views regarding dental curriculums and to determine if there is an impact in student perceptions towards dental education from before and after the “New England Dental Curriculum Hack-a-Thon” event.

## Methods

**Fig. 1:**
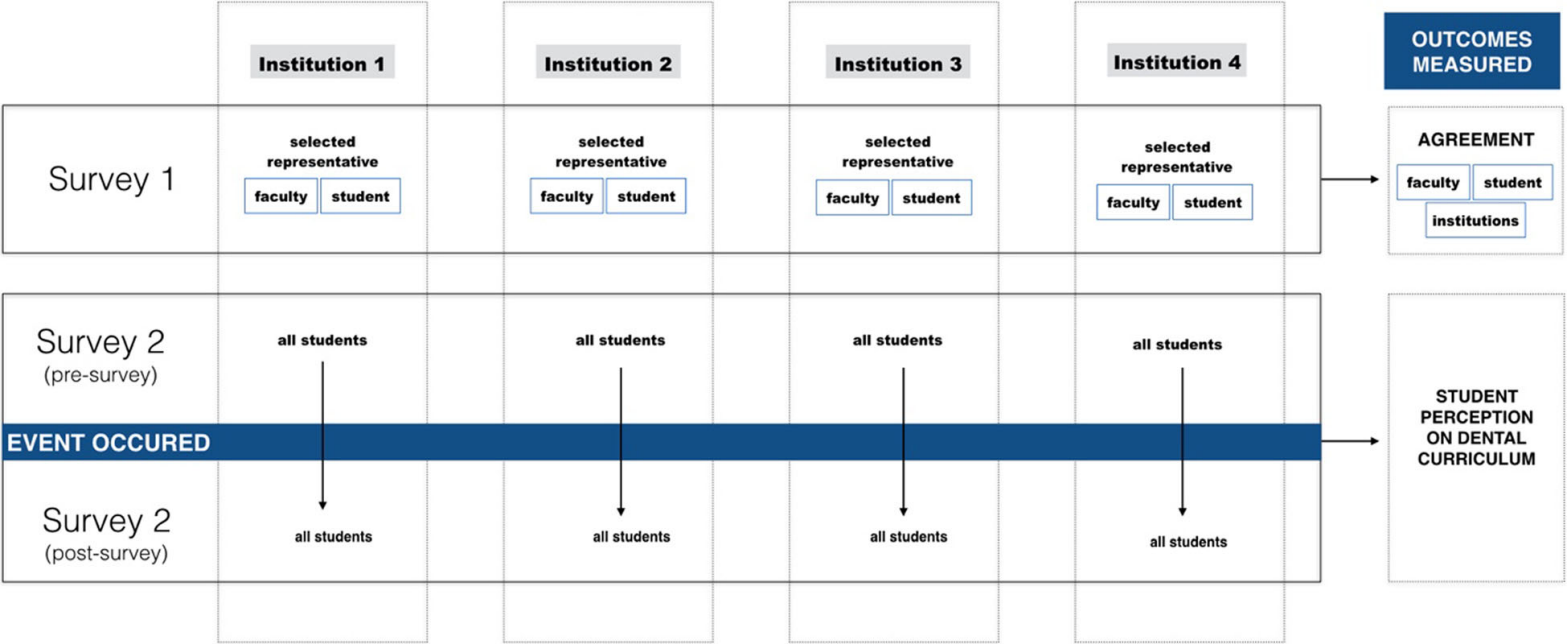
Outline of the proposed study design

This cross-sectional exploratory study design (Fig. 1) received ethical approval from the Tufts Health Sciences Institutional Review Board (#12053). The study participants were dental students and faculty members from four different universities in the New England area: BUGSDM, HSDM, TUSDM, and UNECDM.

Participation or the refusal to volunteer had no effect on a faculty member’s position or on a student’s academic standing. Each school responded to the invitation to participate in the event for the second time in February 2016, as the first event of its kind was held in 2015. Student representatives from the four dental schools, ranging from first through fourth year predoctoral students and postdoctoral residents, attended the conference. Due to the paucity of similar research, we could not conduct a power calculation and therefore decided on a convenience sample for this exploratory study. Based on the event held the previous year, a convenience sample of five students per school was needed in order for each school to be represented in the group assignments. The participants were randomly divided into four groups, with five students from varying classes and schools in each, and asked to create an original dental school curriculum for a fictitious dental school in accordance with the Commission on Dental Accreditation (CODA) standards. Each assigned group compiled a 10-min PowerPoint/Keynote presentation that was presented in front of all the participants of the event. In order to facilitate the discussion among the groups and making sure the tasks were completed, each group was assigned an organizer of the event to act as a student moderator. Faculty members, including Deans and Associate Deans, from the four dental institutions were in attendance and together served as judges for the group presentations. Based on specific criteria, the judges ranked the presentations and an award was given to the team that obtained the highest score.

During the registration process, one co-investigator explained to all the subjects the purpose of the study at the beginning of the event. Two paper-based surveys were distributed: survey 1 to the faculty member representatives and the student representatives from each institution, that evaluated inter- and intra- institutional agreement; and survey 2 to all the dental students, that evaluated the change in perception after attending the event. Participants were asked to fill out the survey 2 before the start of the event (pre-survey) and then the same survey 2 was given after subjects have attended the event (post-survey). An identification code was given to the “pre” and “post” event surveys to be able to compare the data collected at the beginning and at the end of the event. The surveys were designed to describe the format of the dental education and curriculum that dental students have experienced and their thoughts regarding them. Survey 1 was tested for content and construct validity by the authors, after identifying common themes, based on the previous edition of the event and other references in the literature. Survey 2 was adapted from a previously reported survey [17]. Most of the questions were multiple choice, however, a few required a descriptive answer.

A blinded investigator computed the participants’ answers to an excel file. Because of the design of this exploratory study, only descriptive statistics (counts and percentages) were reported.

## Results

Students compiling the four group presentations at the “New England Dental Curriculum Hack-a-Thon” highlighted various topics. Some of the most common topics presented were: decrease lecture time; incorporate more active and modern learning styles i.e. flipped-classroom models and problem based learning; a pass/fail grading system; increase teamwork and peer-to-peer learning; vertical integration; protected elective time for students to pursue individual interests and standardized base curriculum.

Survey 1 was completed by four faculty members (deans and associated deans) and four dental students’ representatives (senior students - leaders of each student group at their respective institution), selected by each individual school. All 14 questions were answered by the student/faculty pairs at a 100% response rate. Responses between student and faculty from the represented institutions were compiled, indicating agreement between faculty and students on their perceptions of dental education. Clinical group practice format was the only topic having the same answer response among all students. Faculty agreed unanimously on basic science integration, evidence based teaching, and support of peer-based teaching. Support of the latest dental technologies and style of dental curriculum only had one institution out of the four have its student/faculty pair in agreement. The only topic to achieve the same answer choice between student and faculty of the same institution was inter-professional integration.

In Survey 2, of the pre-survey respondents, 20 out of the 20 given the survey responded (100%). The post-survey had only had 18 respondents of the 20 responded, (90%). Different answers were reported pre and post-survey among students regarding mandatory attendance in didactic courses (Question 1), electronic based examination preferences (Question 12), and the preference of preclinical courses being held in the first and second years of a four-year dental curriculum (Question 18). A direct comparison of the agreement regarding the results of the pre-survey and post-survey for the questions that were identified can be further reviewed in Table 1.

**Table 1:** Pre- and post-survey data that identifies a change in perception of the participants on different topics (measured on a Likert scale)

Topic	Pre-Survey Median (IQR)	Post-Survey Median (IQR)
Mandatory Attendance	4 (3)	5 (2)
Electronic Examination	3 (2)	2 (2)
Preclinical Courses (D1, D2)	2 (1)	1 (1)

^*^IQR = Correct

^**^Likert scale: 1 = strongly agree, 2 = agree, 3 = neither agree nor disagree, 4 = disagree, 5 = strongly disagree

## Discussion

The concordance of students, faculty and staff members in design and implementation of curricula up to the standards of CODA is integral for the successful training of future dental professionals [16]. Results presented herein reflect the level of agreement on areas of current reform in dental education and highlights the differences in view among students and faculty and across institutions. Current efforts toward education reform discuss the traditional curriculum design wherein a distinct separation exists between basic science/lecture based education and clinical science/patient care. Studies show that adult learners are better able to retain knowledge that has direct application and is integrated into their career objectives [18, 19]. Across all the institutions present at the workshop, faculty agreed that basic science should be delivered in an integrated fashion. This is a departure from a silo approach wherein each subject is taught separately, to a system of multidisciplinary, longitudinal learning. Examples of integrated learning include organ system approach to basic science delivery. Haden et al. showed that while the majority of dental schools present basic science content independently by department, most students prefer to learn this information in an interdisciplinary fashion [20]. While this model is gaining traction among medical schools, dental education has been slow adopt this curricular model and reports suggest that students want to see this change occur [21]. Importantly, integration of basic science principles into clinical instruction is a core principle of CODA standards [18]. The high level of agreement among faculty members representatives at the New England dental schools present at the event, displays the unanimous push to reinforce the science behind clinical care thus leading to better understanding and more knowledgeable dental practitioners and oral health scholars. Relatively high agreement was also seen among students from each institution for the importance of integrating basic science underlining the ubiquity with which this principle has been adopted.

Similarly, faculty across institutions agree that evidence based dentistry are important for the future of dental education. CODA standards require the adoption of evidence-based care in the development of core dental competencies. Adoption of evidenced based clinical care into the core curriculum at dental schools reflects the advancement of dentistry and the push to transform dental care to a more effective/preventative field of medicine. Interestingly, less agreement occurred among students with respect to this topic. Perhaps this reflects a changing tide where the generational expectation is that all care is evidence-based and therefore students do not think that focusing on this area during curriculum development is as important.

Group practice model for clinical instruction displayed high agreement across students and also faculty from each institution. This suggests students’ interest in working in teams, rather than as a solo practitioner, reflecting a national trend in greater amount of dentist working in group practices defined by having greater than three dental practitioners caring for a patient pool. Students now need to be prepared for the communication, leadership and conflict management skills necessary to work in a team-based environment.

Although inter-professional education (IPE) has struggled to gain momentum in dental education, students and faculty members in our project recognize and value the relationship between dentistry and a variety of other fields of study even if they have yet to experience it [22, 23]. IPE involving disciplines and professional students from nursing, medicine, and pharmacy, among others, was viewed as an integral way to prepare for a career as a member of a team of healthcare providers. This approach departs from the common view of private practice dentists existing as a sole service provider. Participants valued a team approach to healthcare delivery and a holistic approach to patient care that begins through IPE in the pre-doctoral curriculum [24].

Bringing students from different dental educational experiences did encourage dialogue and changes in opinion. Results from survey 2 showed that after engaging with a diverse group overall student opinions did change on several topics. Three survey questions resulted in a significant change. Interestingly, after the workshop, mandatory attendance in the didactic portion of the dental curriculum was not seen as important. Self-directed education and inter-class collaboration permeated all of the presentations and are central in many novel teaching methodologies such as flipped classroom. The common themes of the presentations display an overall debate of individualized learning and competition in exchange for a group learning and team-based model of dental education. The low rate of changes in opinion may reflect a desire for change from all students across all schools participating away from individual learning to team based education. Additionally, since the majority of the participants were inherently interested in dental education, they may all have similar views on the direction of curriculum reform regardless of the school which they attend prior to the workshop. Expansion of similar workshops attracting a wider variety of students not directly involved in dental education would provide a robust data set from which to better understand the effect of group work on student perceptions of dental educational reform. In Europe in the 90’s and in the first years of the twenty-first century, there was a great discussion among many dental schools, as part of the DentEd initiative. The meetings were held among the various European universities, including the Schools of Dentistry, especially before and during the site visits in different schools, and compiled students and faculty feedback. An outcome of the Global Dental Education Congress (Dublin, 2007) – was the workshop report compiled by Divaris et al.*,* which suggested the significant value of integrating “student representation and activity under the auspices of reformed International Federation of Dental Education Association (IFDEA)” [25]. Almost 10 years later, this is certainly pertinent in the context of the recently developed initiative “ADEA-ADEE Shaping the Future of Dental Education” (May 2017, London – UK).[26] It should be recognized that the successful adoption of such pedagogical change hinges on the ability of the faculty to meet the set standards [27].

Strengths of this study include the unique setting in which data was collected. To our knowledge, no workshop has been reported wherein students and faculty are brought together to exchange ideas on how best to advance the training of dental professionals. A limitation of this study represents the convenience sample, since the participants were self-interested in dental education. These findings are not generalizable, since the current cohort was representative of the opinions and views of all students/faculty at the participating dental schools, the selection of students and faculty with interest in dental education and positions of leadership related to curricular reform gives an idea of the current trends in curriculum reform. Further studies utilizing representative/generalizable samples should be undertaken to further explore whether the topics presented here hold true across all dental student populations. The current findings could potentially benefit others who might be looking to target specific areas relative to student/faculty perceptions of dental curricula for future research.

## Conclusion

The results presented in this study may provide foundational data for a potential shift in the future of dental education. This could lead to the development of educational research that further examines the relationships between students, faculty, and dental education regulatory organizations with respect to educational reform. Faculty and students have different opinions in traditional educational methods, but the dental curriculum can implement new systems of teaching basic science and applying principles with a clinical approach that mirrors the medical outlook of comprehensive patient care. The results from the New England Dental Curriculum Hack-a-thon is the first of many events that can open the conversation between students, faculty, and dental educational agencies. This event helped validate that intra- and inter-institutional agreement that student perceptions of their dental education requires feedback and change to improve their understanding in the field of the profession. With more open communication and research, studies like this can help progress dental educational reform and the future of dentistry.

## Abbreviations

ADEA: American Dental Education Association
BUGSDM: Boston University Henry M. Goldman School of Dental Medicine
CODA: Commission on Dental Accreditation
HSDM: Harvard School of Dental Medicine
IOM: Institute of Medicine
IPE: Inter-professional Education
TUSDM: Tufts University School of Dental Medicine
UNECDM: University of New England College of Dental Medicine
